# Invasive lobular carcinoma of the breast: the increasing importance of this special subtype

**DOI:** 10.1186/s13058-020-01384-6

**Published:** 2021-01-07

**Authors:** Amy E. McCart Reed, Lauren Kalinowski, Peter T. Simpson, Sunil R. Lakhani

**Affiliations:** 1 UQ Centre for Clinical Research, The University of Queensland, Herston, Brisbane, Australia; 2 QIMR Berghofer Medical Research Institute, Herston, Brisbane, Australia; 3 Department of Histopathology, Sullivan Nicolaides Pathology, Bowen Hills, Brisbane, Australia; 4 Pathology Queensland, Royal Brisbane and Women’s Hospital, Herston, Brisbane, Australia

**Keywords:** ILC, Lobular, Lobular breast cancer, Genomics, Pathology, LCIS, Lobular neoplasia

## Abstract

Invasive lobular carcinoma (ILC) is the most common of the breast cancer special types, accounting for up to 15% of all breast cancer cases. ILCs are noted for their lack of E-cadherin function, which underpins their characteristic discohesive growth pattern, with cells arranged in single file and dispersed throughout the stroma. Typically, tumours are luminal in molecular subtype, being oestrogen and progesterone receptor positive, and HER2 negative. Since last reviewing the lobular literature (McCart Reed et al., Breast Cancer Res 17:12, 2015), there has been a considerable increase in research output focused on this tumour type, including studies into the pathology and management of disease, a high-resolution definition of the genomic landscape of tumours as well as the evolution of several potential therapeutic avenues. There abounds a huge amount of new data, which we will review herein.

## Introduction

**Fig. 1:**
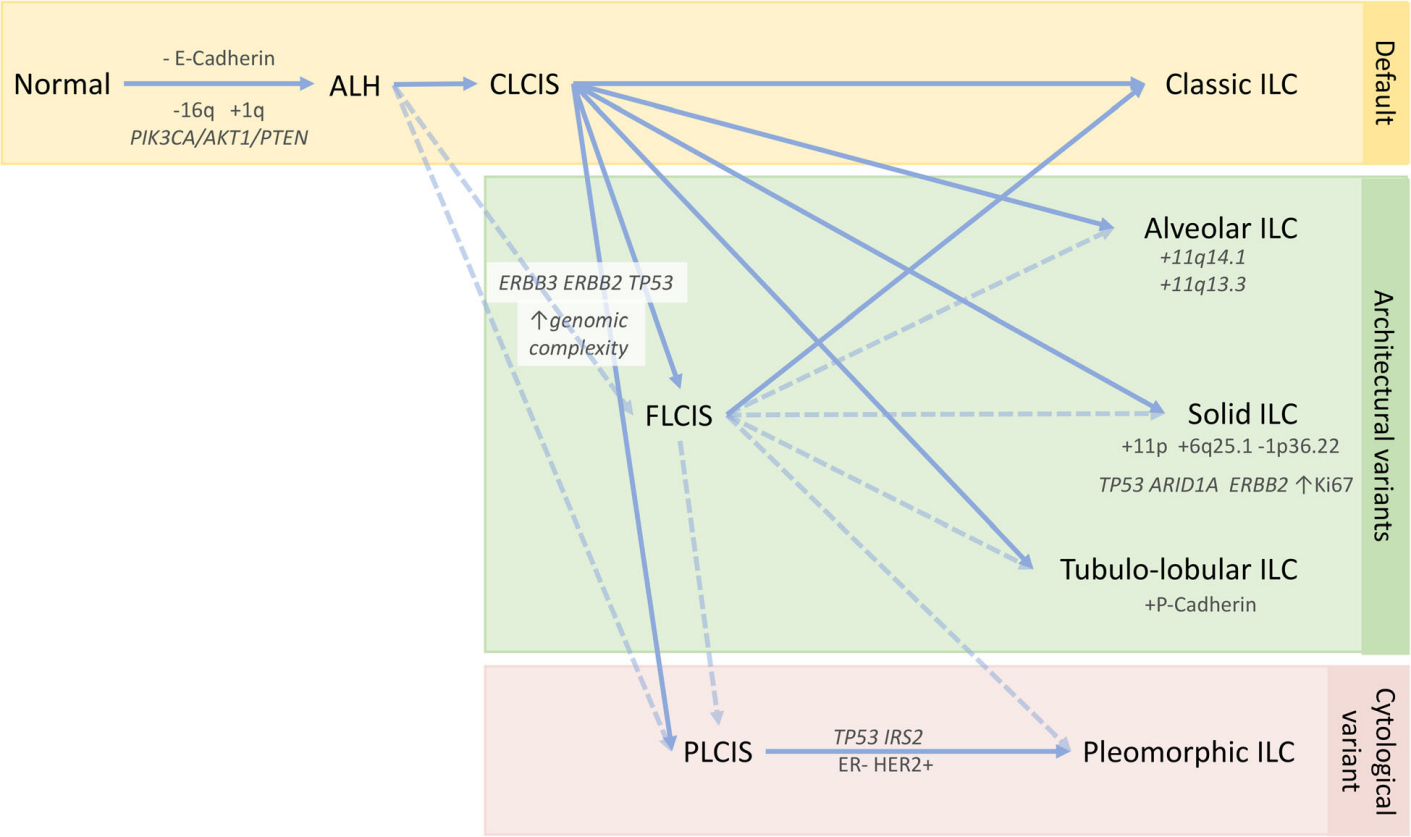
Multistep model of the evolution of classic ILC and its morphological variants. A lineage of ‘lobular’ disease evolves from a normal epithelial cell on a background of a loss of E-cadherin expression and function, and key early somatic alterations involving gain of chromosome 1q, loss of 16q, and mutations in *PIK3CA*, *AKT1*, or *PTEN*. The morphological and molecular diversity of in situ and invasive lobular lesions is likely to be a result of the subsequently arising pattern of molecular alterations that drive progression. Atypical lobular hyperplasia (ALH) is distinguishable from lobular carcinoma in situ (LCIS) based on the extent of proliferation within the lobule. Pleomorphic LCIS (PLCIS) and florid LCIS (FLCIS) can emerge either from ALH (presumably) or from classic LCIS (CLCIS), with an increasing level of genomic complexity and the accumulation of mutations in driver genes such as *ERBB2*, *ERBB3*, and *TP53*. Various morphological variants of ILC have also been described (see also Fig. 2), which exhibit either architectural or cytological atypia relative to the classic invasive type, which we imagine being the ‘default’ pathway of evolution. A number of important points to note: (1) the genomic alterations listed may arise during any stage of progression, though are likely to be acquired at the in situ stage, or earlier (e.g. amplification of 11q13 is evident in the in situ stage); (2) it is assumed FLCIS may progress to alveolar, solid, tubulo-lobular variants, or even the pleomorphic type; (3) it is uncommon for invasive tumours to be of a pure variant morphology, with tumours often also exhibiting classic and/or other variant patterns; (4) a variety of molecular alterations have been associated with some of these morphological variants, but these are not necessarily pathognomonic of the architectural variant; and (5) the interplay between the malignant cells and extracellular matrix may also impact the resulting growth pattern. -, loss; +, gain; dotted line, anticipated route of progression; solid line, demonstrated route of progression

Invasive lobular carcinoma is the most common ‘special’ histological subtype of invasive breast carcinoma. From an evolutionary point of view, these tumours arise from a family of non-obligate precursor lesions called atypical lobular hyperplasia (ALH) and lobular carcinoma in situ (LCIS), which may be collectively termed lobular neoplasia (LN). Even within this narrow spectrum of pre-invasive lesions and frank invasive carcinoma, there is significant morphological and biological heterogeneity. The multistep model of breast cancer progression [1, 2] contends that although lobular carcinomas arise along the low-grade, ER-positive arm of the pathway (with low-grade, ER-positive ductal lesions), de-differentiation to higher grade lesions can occur through acquisition of alterations in oncogenes such as *ERBB2* and *TP53*, producing a spectrum of heterogenous proliferations (Fig. 1).

Lobular neoplasia are mostly an incidental finding and comprise neoplastic proliferation of characteristically discohesive cells which fill and distend the terminal duct lobular units. LN encompasses both ALH and LCIS, and the boundary between the two is defined by an arbitrary cut-off using a quantitative measure, depending on the relative extent of involvement of the terminal duct lobular unit (TDLU); if more than 50% of the TDLU is occupied, the lesion is upgraded to LCIS. LN is considered to be a non-obligate precursor of invasive cancer, with ALH associated with a 4–5 times increased relative risk for subsequent cancer, and LCIS an increase of 8–10 times the risk; the risk is bilateral but predominates for the ipsilateral breast [3]. The clinical and morphological features of LCIS and its morphological variants have recently been extensively reviewed elsewhere and will not be covered herein [4, 5].

Classic invasive lobular carcinoma (ILC) typically demonstrates single cell infiltration and a characteristic targetoid pattern of growth with minimal associated stromal response [3] (and reviewed in [6]). This pattern of subtle invasion is such that the size of the tumour often exceeds the imaging findings and obtaining clear surgical margins may be challenging. Although ILCs are generally palpable, a high false-negative mammography rate is possible (in 19–43%; reviewed in [7]). In addition to the classic form of ILC, which is typically histological grade 2, there are special morphological subtypes including Pleomorphic, Solid, Alveolar, and Tubulo-lobular [3, 8–12]. These variants are rarely seen as pure forms and are more likely to be present with the classical type. ILC and its subtypes are typified by a loss of cellular adhesion, frequently the result of biallelic inactivation (i.e. gene mutation combined with gene deletion) of the *CDH1* gene encoding E-cadherin, although other mechanisms of expression loss also feature. ILCs are normally oestrogen (ER) and progesterone (PR) receptor positive, and as such patients are indicated for hormone therapy. Whilst the biological characteristics of ILC afford patients a good prognosis in the short term, it has become clear that the longer-term prognosis of ILC is frequently worse than for patients with the more commonly diagnosed invasive breast carcinoma of no special type (IBC-NST; invasive ductal carcinoma, IDC) (reviewed in [6]).

The metastatic presentation of ILC has long been considered unique [13, 14], with a predilection for common sites (liver, lung, bone), but also gastrointestinal and gynaecological sites of colonisation [15, 16]; recent studies further support this. Inoue et al. showed that lung metastases were less prevalent, but peritoneal metastases are significantly higher in ILC (assumed predominantly classic ILC) compared to ER-positive IBC-NST [17]. A recent study of metastatic spread to gynaecological sites demonstrated an association with ILC and young age at diagnosis and confirmed earlier reports of the wide metastatic colonisation of ILC [18]. Immunophenotyping showed a heterogeneous interplay between hormone receptors and their co-factors during progression, including frequent downregulation of PR expression and variable changes between AR, GATA3, and FOXA1 seen in different metastases within the same patient [18]. Rarer presentations are increasingly being published in the literature, further highlighting the peculiar natural history of ILC. For example, numerous case reports of ILC seeding as orbital metastases appear to suggest these are more likely to arise from an ILC than other types, and in a sole example of a mixed ductal-lobular carcinoma, only the lobular component was found in the orbital metastasis (e.g. [19–22]).

In the last 5 years, an impressive body of work on ILC has amassed. There abounds a huge amount of new data, including studies into the pathology and management of disease, the genomic landscape of ILC and in particular somatic alterations associated with therapy resistance, and the evolution of several potential therapeutic avenues, which we will review herein.

## What is new in the phenotypic and molecular characteristics of lobular carcinoma in situ?

**Fig. 2:**
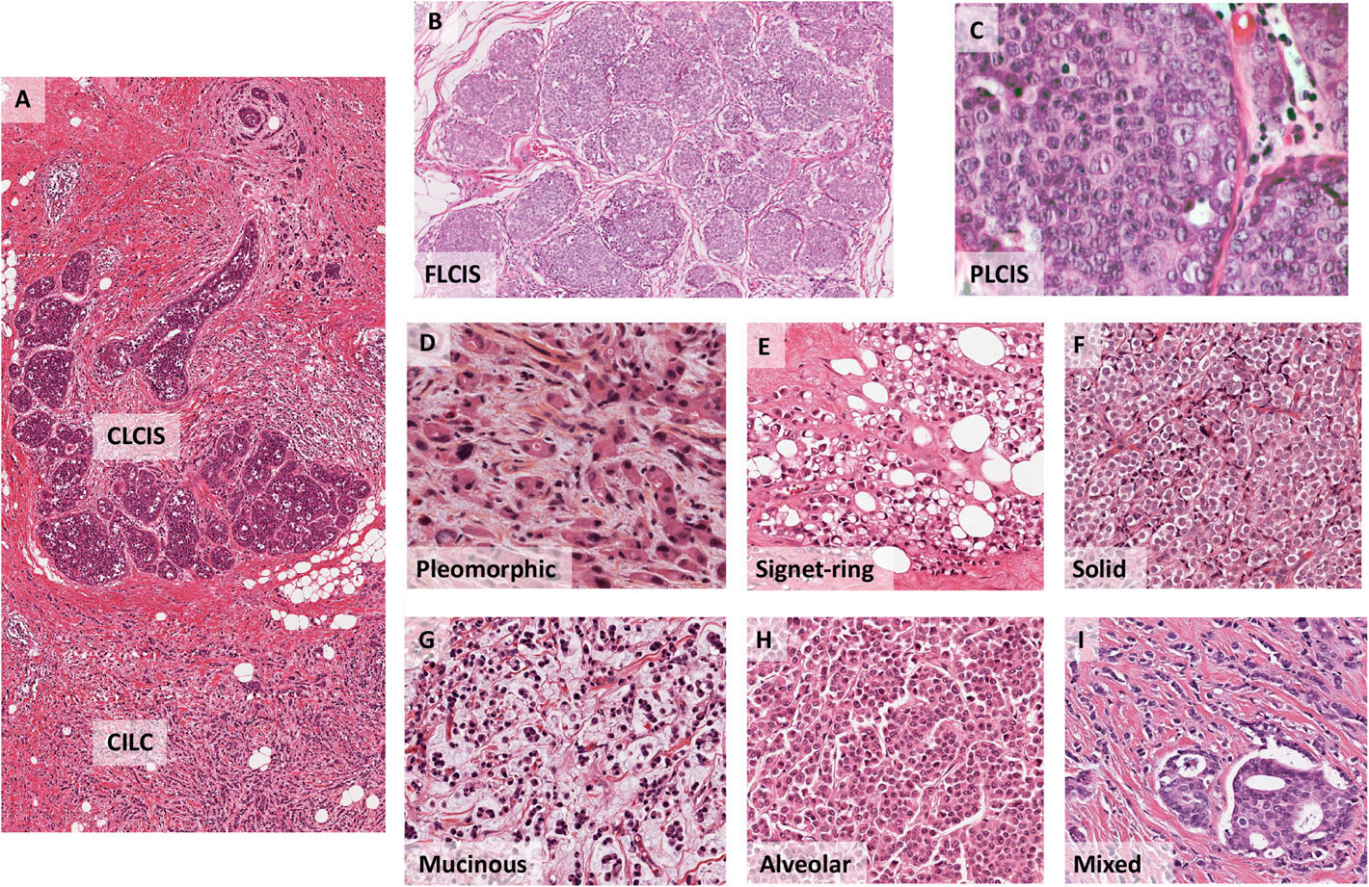
Histological examples of lobular variants. **a** CLCIS and CILC (as marked), × 40 magnification. **b** FLCIS shows the cytology of CLCIS with marked distention of lobular units to form a confluent mass-like lesion, × 40 magnification. **c** PLCIS with cytological atypia—nuclear pleomorphism with large vesicular nuclei and nucleoli—at least some × 4 the size of lymphocytes; × 600 magnification. **d** PILC, characteristic discohesion but with high-grade pleomorphic nuclei, with pink, foamy cytoplasm typical of an apocrine phenotype, × 400 magnification. **e** ILC with signet ring cell morphology, × 200 magnification. **f** Solid with sheets of classic type cells, × 200 magnification. **g** ILC showing mucinous/histiocytoid morphology, × 200 magnification. **h** Alveolar variant with cluster/globular arrangement of at least 20 cells, × 200 magnification. **i** Mixed ductal-lobular carcinoma, × 200 magnification. The variants of ILC rarely present in pure form and are more likely to occur as mixed lesions with classic type and/or other subtypes, e.g. classic, pleomorphic, and solid. CILC, classic ILC; CLCIS, classic LCIS; FLCIS, florid LCIS; PLCIS, pleomorphic LCIS

The WHO Classification [3] recognises three variants of LCIS: classic (CLCIS), pleomorphic (PLCIS), and florid (FLCIS) (Fig. 2). The defining features of both PLCIS and FLCIS have recently been clarified: PLCIS is characterised by cells with enlarged nuclei (4× size of lymphocytes) or similar cytological features to those seen in high-grade ductal carcinoma in situ (DCIS) [3]. FLCIS is characterised by confluent expansive growth, and there must be marked distension of involved acini with little intervening stroma or an expanded acinus or duct approximately 40–50 cells in diameter [3]. PLCIS is therefore characterised by its degree of cytological atypia, whereas FLCIS describes an architectural pattern (confluent, mass-like growth) with proliferation that is of classic type (CLCIS). Unlike CLCIS, PLCIS and FLCIS are more likely to have comedo-necrosis and calcifications and hence clinical and radiological presentations [23, 24]. CLCIS is invariably ER and PR positive, and HER2 negative; FLCIS exhibits a similar phenotype, though may occasionally be HER2 positive, whilst PLCIS exhibits a more varied phenotype, with less frequent hormone receptor positivity, and an increased likelihood for HER2 overexpression, particularly in the apocrine-type of PLCIS as well as a higher proliferative index [23, 25–27]. The natural history of PLCIS and FLCIS is as yet not well understood, and as such, relative risk of progression to frank invasive disease remains unclear, and we await long-term outcome data [4, 28].

An accumulating volume of work has investigated the molecular characteristics of CLCIS and these special subtypes. The application of genomic technologies (copy number profiling, whole exome and targeted panel sequencing) to a large set of LCIS confirmed that these lesions were frequently clonally related to other more malignant lesions found to co-occur in the same specimen (i.e. DCIS and ILC), and that intralesion molecular heterogeneity was also identified within LCIS, particularly among those lesions clonally related to DCIS and/or ILC [29, 30]. The data further supports the idea (i) that LCIS shares molecular characteristics with its invasive counterpart suggesting they do indeed have a common clonal origin and that LCIS is a non-obligate precursor of ILC, and (ii) that considerable genomic diversity may arise in LCIS whilst the lesion is still confined within the ductal architecture accounting for some of the morphological and biological variability observed on the progression to invasive cancer [29, 30].

Some of this intralesional LCIS heterogeneity accounts for the occurrence of PLCIS and FLCIS. There is a striking similarity in the genomic profiles of CLCIS, PLCIS, and FLCIS (and invasive tumours), with recurrent gains of 1q and losses on 16q and *CDH1* mutations suggesting they arise from a common aetiology (Fig. 1). FLCIS and PLCIS with apocrine differentiation are more genomically complex than CLCIS and non-apocrine PLCIS, with an increase in the number of amplifications, genomic losses, and breakpoints [25, 26]. An increased frequency of *ERBB2*/HER2 mutations or amplifications has been reported in PLCIS compared to classic LCIS [31]. Exome sequencing of a small cohort of PLCIS and two cases of FLCIS demonstrated frequent alterations similar to those seen in classic LCIS and ILC, including 16q loss, 1q gain, and mutations in *CDH1*, *PIK3CA*, *RUNX1*, and *CBFB* [27, 32] (Table 1). However, there was a striking difference within the special variants of LCIS, with highly recurrent *ERBB2* and *ERBB3* alterations (mutations or amplifications) present in 94.7% of cases studied [27]. The reported missense mutations or insertions predominantly affected the tyrosine kinase domains of the epidermal growth factor family of receptors, suggesting that these genes may be drivers of oncogenicity in these special LCIS subtypes [27]. This important finding is supported by another study that also demonstrated an enrichment of *ERBB2* and *ERBB3* mutations in 50% of PLCIS and FLCIS variants relative to co-occurring CLCIS [33]. Thus, between 50 and 94% of PLCIS and FLCIS harbour *ERBB2* or *ERBB3* mutations (Table 2). The presence of common mutations shared between lesions within an individual case inferred that PLCIS or FLCIS had a common clonal ancestry to classic LCIS, but that the additional acquisition of mutations in *ERBB2* and *ERBB3* (as well as in *TP53*, *CCND1* and increased copy number aberrations) [33] suggests these alterations are likely drivers of the enhanced cytological atypia and proliferative state seen in these variants. Overall, the biology of the lobular neoplasia is becoming clearer, and the future may bring detailed assessments of relative risks of morphological variants and answer challenges around managing pre-invasive lesions, important considerations given the role of routine mammographic screening in their identification.

**Table 1:** Somatic mutations in lobular neoplasia, primary ILC (and their variants), and metastatic ILC

		Lee et al. [29]	Harrison et al. [27]	Shamir et al. [33]	Ciriello et al. [32]	Michaut et al. [34]	Desmedt et al. [35]	Rosa-Rosa et al. [36]	Zhu et al. [37]	Richard et al. [38]		Pareja et al. [39]		Sokol et al. [40]
		CLCIS (*n* = 43)	PLCIS (17) FLCIS (2) (*n* = 19)	PLCIS (10) FLCIS (6) (*n* = 16)	ILC (*n* = 127)	ILC (*n* = 144)	ILC (*n* = 413)	PILC (*n* = 27)	PILC (*n* = 17)	Primary ILC (*n* = 32)	Metastatic ILC (*n* = 32)	Primary ILC (*n* = 127)	Metastatic ILC (*n* = 132)	Metastatic ILC (*n* = 180)*
***Mutations***	***CDH1***	81.0%	94.7%	94.0%	63.0%	42.7%	65.4%	89.0%	59.8%	53%	62.5%	82%	76%	77%
	***PIK3CA***	32.0%	31.6%	56.2%	48.0%	33.8%	43.3%	33.0%	52.9%	44%	44%	57%	52%	52.9%
	***ERBB2***	7.0%	68.4%	37.5%	4.0%	4.3%	5.1%	26.0%	17.6%	12.5%	15.6%	2%	12%	8.3%
	***ERBB3***	NR	21.1%	18.8%	NR	2.9%	3.6%	NR	23.5%	3%	0%	NR	NR	< 5%
	***RUNX1***	2.3%	21.1%	NR	10.0%	NR	3.4%	NR	11.8%	3%	6%	9%	5%	~ 7%
	***CBFB***	19.0%	15.8%	12.5%	2.0%	NR	NR	NR	17.6%	NR	NR	NR	NR	NR
	***FOXA1***	4.7%	10.5%	31.3%	7.0%	NR	9.0%	NR	5.9%	15.6%	15.6%	8%	11%	NR
	***GATA3***	4.7%	10.5%	12.5%	5.0%	5.1%	7.3%	7.0%	5.9%	15.6%	15.6%	3%	7%	2.2%
	***ARID1A***	2.3%	10.5%	6.3%	17.0%	7.0%	6.3%	15.0%	5.9%	12.5%	12.5%	8%	11%	~ 12%
	***TP53***	0%	5.3%	18.8%	8.0%	3.6%	7.3%	19.0%	11.8%	18.7%	9%	9%	20%	23.9%
	***TBX3***	9.3%	NR	NR	9.0%	8.0%	13.3%	7.0%	23.5%	21.8%	18.7%	10%	16%	12.8%
	***KMT2C***	2.3%	NR	NR	7.0%	10.0%	8.0%	19.0%	35.3%	NR	NR	NR	NR	< 5%
	***MAP3K1***	4.7%	NR	NR	6.0%	5.1%	5.0%	19.0%	35.3%	18.7%	15.6%	10%	10%	< 5%
	***ESR1***	NR	NR	NR	NR	1.4%	NR	NR	5.9%	12.5%	15.6%	2%	15%	17%
	***AKT1***	4.7%	NR	NR	2.4%	5.1%	4.1%	7.0%	5.9%	6.2%	9.4%	NR	NR	~ 5%
	***PTEN***	0%	NR	6.2%	7.0%	1.4%	3.9%	NR	0%	3.1%	0%	9%	9%	~ 10%
	***NF1***	NR	NR	0%	NR	4.3%	1.0%	7.0%	24%	3.1%	6.2%	2%	8%	12.2
***Amp***	***CCND1***	NR	26.3%	18.8%	17.0%	15.0%	38.0%	11.0%	12.0%	33.3%	38.0%	17%	17%	22%
	***ERBB2***	4.7%	31.5%	12.5%	7.0%	4.0%	0.0%	4.0%	6.0%	33.3%	15.6%	2%	5%	NR
	***FGFR1***	0%	0%	NR	NR	NR	25.3%	7.0%	23.5%	6.2%	6.2%	7%	11%	7.8%

*CLCIS* classic LCIS, *FLCIS* florid LCIS, *NR* not reported, *PILC* pleomorphic ILC, *PLCIS* pleomorphic LCIS

*Frequencies of some alterations estimated from Figure 2 in Sokol et al. [40]

**Table 2:** *ERBB2* and *ERBB3* mutations in different stages of lobular neoplastic progression

Study		***ERBB2*** alteration frequency			***ERBB3*** alteration frequency			Notes
		*Total %*	*Mut*	*Amp*	*Total %*	*Mut*	*Amp*	
**In situ**	Harrison et al. [27]	94.7%	13/19	6/19	21%	4/19	0/19	17 PLCIS; 2 FLCIS
	Shamir et al. [33]	50%	6/16	2/16	18.7%	3/16	0/16	10 PLCIS; 6 FLCIS; *ERBB2* and/or *ERBB3* alterations in 60% PLCIS and 50% FLCIS
**Primary ILC**	Zhu et al. [37]	17.6%	3/17	–	23.5%	4/17	–	PILC
	Rosa-Rosa et al. [36]	26%	7/27	1/27	–	–	–	PILC; association with nuclear grade 3
	Christgen et al. [41]	5%	5/106	–	–	–	–	Grade 3 but no association with solid or pleomorphic
	Cao et al. [42]	19%	–	13/70	–	–	–	Amplification; no mutation assessment
	Deniziaut et al. [43]	15%	6/55	–	0%	0/55	–	Grade 3; positive association with solid presentation
	Ping et al. [44]	6%	*6/100*	*–*	*–*	*–*	*–*	*CDH1* altered with *ERBB2* mutation correlates with poor prognosis
	Lien et al. [31]	52.2%	5/24	8/24	–	–	–	PILC; 2% in classic ILC
**mILC**	Ma et al. [45]	7.8%	4/51	–	–	–	–	Metastatic ILC; confirmed neratinib efficacy in *ERBB2* mutants in phase II trial; detection in ctDNA
	Ross et al. [46]	22.7%	4/22	1/22				Relapsed ILC; *ERBB2* mutation enriched in *CDH1* mutant tumours; I gene fusion not tabulated (*ERBB2*–*GRB7*)

*CILC* classic ILC, *ctDNA* circulating tumour DNA, *FLCIS* florid LCIS, *mILC* metastatic ILC, *PILC* pleomorphic ILC, *PLCIS* pleomorphic LCIS

## What is new in invasive lobular carcinoma?

### Refining the histopathology of ILC

Classic ILCs typically show a luminal A molecular phenotype with around 90% of cases showing strong oestrogen receptor (ER) positivity together with 60–70% of cases also exhibiting strong progesterone receptor (PR) expression (rates that are significantly higher compared to that seen in IBC-NST [15, 47]); they are usually negative for human epidermal growth factor receptor 2 (HER2) gene amplification and overexpression (Fig. 2) [3, 15, 47]. It has long been recognised, however, that an important subpopulation of cases do not conform to this ER/PR+, HER2− phenotype and as such are either ER/PR negative, triple negative, or HER2+, with high grade and the pleomorphic ILC (PILC) subtype more likely than other morphological subtypes to exhibit such phenotypes [23, 47–51]. A recent study of Mexican breast cancer patients compared the disease-free survival and overall survival between ILC and IBC-NST. The authors showed the overall survival in both triple-negative ILC and HER2+ ILC was significantly worse compared to their IBC-NST counterparts raising the possibility that within ILC, HER2+ status or triple-negative status identifies clinically important subtypes of ILC [52, 53]. A comparison of patients with HER2+ ILC and HER2+ IBC-NST provided further evidence that HER2+ ILC has different clinical and biological characteristics [54]: relative to HER2+ IBC-NST, HER2+ ILCs were more often multicentric or multifocal, with a lower histological grade and proliferative index, and show more frequent nodal metastases (i.e. these are similar features observed when ILC is compared to IBC-NST, regardless of HER2 status [15, 47, 55, 56]). Whilst HER2+ ILC and IBC-NST have differing characteristics, both groups appear to benefit similarly from adjuvant treatment with trastuzumab, with similar recurrence rates, indicating HER2+ ILC patients do benefit from anti-HER2 therapy [54, 57].

**Fig. 3:**
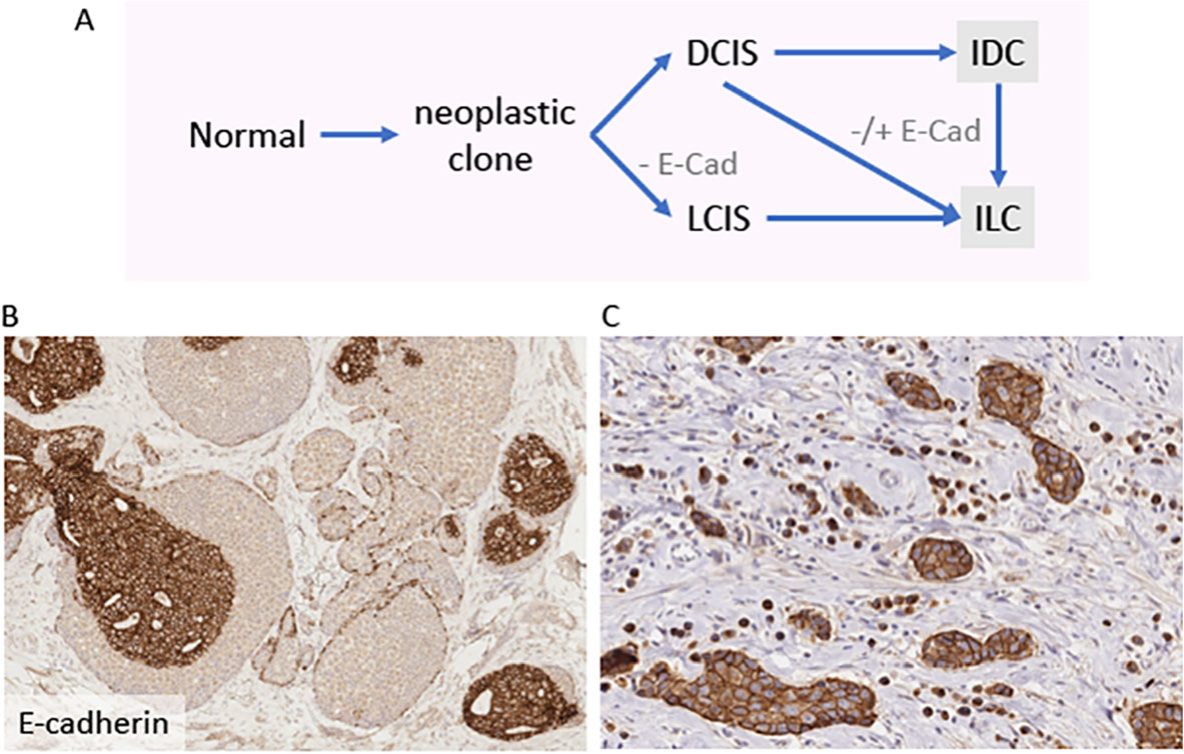
Multistep model of evolution of tumours with morphological features indicative of mixed ductal and lobular carcinomas. **a** Co-existing lesions with both ‘ductal’ and ‘lobular’ morphology are frequently clonally related, suggesting shared origins of a common neoplastic clone. Early divergence leads to the co-occurrence of LCIS and DCIS, and in such cases LCIS and associated ILC are likely to be negative for E-cadherin. Tumour cells exhibiting a lobular pattern of growth can also emerge from the ‘ductal’ pathway, and in such cases E-cadherin might be positive or aberrantly expressed. Modified from McCart Reed et al. [58]. Immunohistochemical staining for E-cadherin in different tumours: **b** × 10 magnification showing co-existing E-cadherin-positive DCIS and E-cadherin-negative LCIS; **c** showing strong membrane E-cadherin positivity in tumour cell nests and aberrant (cytoplasmic) E-cadherin staining in adjacent single cells. DCIS, ductal carcinoma in situ; LCIS, lobular carcinoma in situ; IDC, invasive ductal carcinoma; ILC, invasive lobular carcinoma; - E-cad, E-cadherin loss; -/+ E-cad, variable expression (loss, positive, aberrant)

ILC can be seen in a mixed growth pattern together with other types of invasive carcinoma, most frequently IBC-NST, in around 5% of all breast cancer cases; these are variably referred to as mixed ductal-lobular carcinomas, invasive ductal carcinoma with lobular features, invasive ductulolobular carcinoma, or mixed IBC-NST and invasive lobular carcinoma [3]. Such tumours represent an important example of intra-tumour, morphological heterogeneity, which is probably direct evidence of underlying intra-tumour clonal heterogeneity at the molecular level. Examination of E-cadherin expression in these mixed tumours has shown that the ‘ductal’ component typically shows normal membranous expression, whilst the ‘lobular-like’ morphological growth pattern may show complete loss of staining akin to that seen in ILC, but is most likely to exhibit positive and/or aberrant staining (Fig. 3) [58–60]. Furthermore, analysis of the disparate morphological components by whole exome sequencing demonstrated that all components were related to a common neoplastic clone. A modification to the progression pathway was proposed, wherein a lobular phenotype can emerge from an evolving ductal lineage following the loss of functional cellular adhesion (Fig. 3) [58]. A number of studies have compared the clinicopathological features of mixed tumours to pure ILC and/or IBC-NST [58–64]. In all datasets, mixed tumours are most frequently grade 2 and of an ER/PR-positive, HER2-negative phenotype; although like ILC, some tumours may be high grade, ER negative, and/or HER2 positive. Several studies show the rates of breast cancer specific survival and disease-free interval are similar between mixed tumours and pure ILC and/or IBC-NST. Interestingly, recent data suggest outcomes were worse in ILC compared to mixed tumours in postmenopausal women, but not in premenopausal women, and treatment with aromatase inhibitors (as monotherapy or sequentially with tamoxifen) was better than tamoxifen alone [63].

The capacity of neoplastic cells of ILC to create tubular structures in the absence of E-cadherin-facilitated cellular adhesion has long intrigued researchers. Recently, a series of ILC with tubular elements representing the rare tubulo-lobular variant of ILC was investigated. The authors demonstrated the phenomenon of focal cadherin switching, wherein activation of P-cadherin, in an otherwise E-cadherin-negative tumour, rescued the function of the adherens junction enabling tubule formation [65]. Notably, co-incident LCIS did not express P-cadherin. A recent case report also described a new variant of lobular carcinoma mimicking an encapsulated papillary carcinoma but lacking expression of E-cadherin [66], further testament to the understudied nature and appreciation of morphological heterogeneity in ILC. It is increasingly clear that the spectrum of disease even within the lobular category is broad, and whilst we speak to ILC as being ER positive, and of a relatively homogeneous histology, once again the extent of diversity within breast cancer is surprising.

### The immune microenvironment of ILC

The emerging field of immuno-oncology has precipitated standardisation of the assessment of tumour infiltrating lymphocytes (TILs) [67]. Long considered to be immune ‘cold’ (limited TILs), two recent studies [68, 69] showed that TIL levels were indeed lower than those observed in IBC-NST, but nevertheless that a proportion of ILC elicit high TILs that likely impact the behaviour of the tumour. Desmedt and colleagues demonstrated high numbers of TILs (> 10%) were present in approximately 15% ILC cases (cohort contained 50% classic ILC and 50% ILC variants), and that this was associated with young age at diagnosis, positive lymph nodes, increased proliferation, and ultimately with a poorer prognosis [68]. The same group recently examined a cohort of matched ILC and metastases (EuroILC cohort) for TILs and genomic features (see below) [38]. TILs were assessed by the same pathologists as in [68]; primary tumours from the EuroILC series with matched metastases had significantly lower levels of sTILs than the primary tumours in the original study [68], whilst paired primary and matched metastases had no difference in their levels of TILs. As before, cases with higher sTILs were also associated with younger age at diagnosis, and with mixed, non-classic histologies. The sTIL assessment of the matched metastases showed that the sTIL infiltration was unrelated to metastatic site [38]. In another large study, TIL content was categorised into 3 groups: no TILs (*n* = 239), ≤ 5% TILs (*n* = 185), and > 5% TILCs (*n* = 39 [69];), with a mean TIL score of 2.7%. Here, TILs could stratify poor outcome independently of lymph node status or molecular subtype of ILC. Furthermore, molecular subtyping of ILC by transcriptomic analyses has shown enrichment of specific immune signatures in a subset of ILC, including for *STAT5 alpha*, *PD-1*, *PD-L1*, and *CTLA4* mRNAs, and that this may impact patient outcomes [32, 34, 70].

Recent evidence suggests that *PIK3CA* mutation status may impact the tumour immune microenvironment in ER-positive breast cancer [71], and this is therefore likely to be important in ILC. As noted by Oesterreich and colleagues [72], the emerging data for ILC in the immune-oncology space is providing an excellent foundation for future trials and exploration of novel compounds in ILC treatment.

### The genomic landscape of ILC and its morphological variants

Recent large, landmark studies have built on previous foundational molecular studies in ILC to comprehensively define the pattern of somatic mutations and structural alterations present in primary ILC [32, 34, 35]. Despite increasing numbers of samples profiled across a number of high impact studies including from TCGA and RATHER cohorts, the frequency of *CDH1* (and other gene) mutations in ILC is variably reported (e.g. 43–80% of cases; Table 1). It is interesting to note that in studies examining LCIS, in which microdissection was employed to enrich for neoplastic cells, the frequency of *CDH1* mutation is 81–94%, suggesting that some of this variability may be related to the sensitivity of sequencing platforms used when analysing a tumour type with a diffuse growth pattern and hence samples of potentially low tumour cellularity.

In addition to the well-characterised alterations in *CDH1*, and the copy number alterations involving gain of chromosome 1q, loss of 16q, and amplifications of 8p12 (*FGRF1* locus) and 11q13 (*CCND1* locus), the importance of mutations in driver genes *PIK3CA*, *PTEN*, *AKT1*, *TBX3*, *FOXA1*, and *ERBB2/3* is repeatedly observed (Table 1). Whilst *CDH1* mutations are pathognomonic for ILC and the morphological variants, there are other genes which are altered to different frequencies in ILC compared to IBC-NST. One notable finding is that *FOXA1* and *GATA3* mutations are reciprocally more common in ILC or IBC-NST, respectively [32, 35]. Further, *PIK3CA*, *PTEN*, and *AKT1* are collectively mutated in over half of all ILC and at a higher frequency to molecular subtype-matched (luminal A) IBC-NST, thus leading to an enriched Akt pathway activation in ILC [32, 35]. Also of note, the frequency of mutations in, for example, *TP53*, *ESR1*, and *ERBB2* increases with increasing severity of disease, for example CLCIS vs. FLCIS/PLCIS (as noted above), CILC vs. PILC, or primary vs. metastatic ILC (Table 1**,** and see below), attesting to the importance of these gene alterations in driving a more aggressive tumour biology which, importantly, are linked to endocrine therapy resistance.

Morphological variants of ILC show the same overall pattern of alterations to classic type, yet with some notable additions that likely underlie the different histologic patterns of growth. As discussed above, *ERBB2* and *ERBB3* mutations are more commonly identified in PLCIS than classic LCIS, and this holds true for their invasive counterparts classic and pleomorphic ILC [33, 36]. This also presents compelling evidence that PLCIS/PILC may evolve from CLCIS/CILC with the acquisition of such additional genetically complex changes, similar to that previously observed when comparing the genomic landscape of classic and pleomorphic ILC [73] (Fig. 1). An enrichment of *IRS2* mutations has also been reported in approximately 30% of PILC tumours, with in vitro studies suggesting a role in enhanced invasion [37].

As well as characterising the overall genomic features of ILC, Desmedt et al. [35] defined interesting mutation and copy number alterations with different ILC variant morphology: solid ILC variants were shown to be enriched for *ERBB2*, *TP53*, and *ARID1A* mutations; 11p and 6q25.1 (*ESR1*) gains; and 1p36.22 (*ARID1A*) deletions, whilst the alveolar variant harboured 11q13.3 (*CCND1*) and 11q14 (*PAK1*) gains (see Fig. 1); mixed, non-classic types had mutations in *TP53* and *ERBB2*. Clinically, the two most important morphological variants of ILC are the classic and pleomorphic types. The other types collectively exhibit a worse prognosis compared to classic ILC [74], perhaps related to the accumulation of these additional driver alterations; however, morphologically cases with variant morphology frequently exhibit mixed appearance with classic and/or other growth patterns. A large study separately dissecting different ILC growth patterns and correlating with genomic features (as was illustrated above regarding CLCIS, PLCIS, and FLCIS) has yet to be undertaken.

### Mutational drivers of therapy resistance in ILC progression

The genomic mechanisms underlying therapy resistance [75] are a burgeoning field and are already producing important data and insights into the clinical management of ILC relapse. *ESR1* mutations are known to occur in the hormone therapy resistance setting, and the prevalence and type of *ESR1* mutation in ILC are comparable to IBC-NST [40, 76]. The increasing importance of *ESR1* copy number gains is emerging, with gains and amplifications reported in 14% and 10% of ILC cases, respectively; this was significantly associated with disease recurrence [42]. *ESR1* copy number gains in metastatic ILC were significantly enriched in bone metastatic deposits [77].

A formative genomics study by Razavi and colleagues was conducted on prospectively collected advanced ER-positive breast cancers, including on post-treatment biopsies in the neoadjuvant or metastatic settings [78]. The authors used the MSK-IMPACT targeted gene panel sequencing assay, and they confirmed interesting histo-specific changes enriched in ILC (relative to IBC-NST) including *TBX3* (N297) and particular mutations in the forkhead domain of *FOXA1*, with an enrichment of *ERBB2* and *NF1* mutations in metastatic clones [78]. These findings are independently supported by others [40, 79], with both *NF1* and *ERBB2* mutations being enriched in metastatic ILC; indeed, both gene mutations are also found to be mutually exclusive with *ESR1* mutations [39, 78], suggesting they play important roles in mediating endocrine therapy resistance in both ER-positive breast cancer overall and specifically in ILC.

The acquisition of *ERBB2* and *ERBB3* mutations in ILC has been shown to be associated with an increased risk of relapse and poorer outcomes, likely representing an escape mechanism to endocrine therapy [44]. Indeed, a recent, large meta-analysis showed that ILCs account for 47% of all *ERBB2* mutated (not amplified) cases, and that targetable *ERBB2* mutations are an independent prognostic marker of poorer 10-year overall survival [80]. This team then postulated that mutation of *ERBB2/3* may initiate alternate downstream activity (as opposed to *ERBB2/3* amplification) and derived a gene expression signature to measure this. This signature measured the impact and actionability of the *ERBB2* alterations, and exhibited potential clinical value by being predictive of neratinib response in breast cancer cell line data. As most of the mutations in *ERBB2* and *ERBB3* cause activation of the HER2 pathway, they may be amenable to treatment with HER2 inhibitors (e.g. neratinib, lapatinib) [44, 81]. However, it should be noted one of the most common recurrent *ERBB2* mutations, p.L755S, confers resistance to lapatinib [82]. The frequency of alterations in *ERBB2* in lesions with lobular morphology is summarised in Table 2.

Mutation data from the Razavi et al. [78] was recently subjected to re-analysis by Pareja et al. [39] and was also used by Richard et al. [38] as a comparator to an independent series of primary and metastatic ILC sequenced from the EuroILC cohort, including paired primary and metastases from the same patient. In support of the role of several gene mutations in therapy resistance and progression [75], Richard et al. demonstrated that 41% (in MSK-IMPACT cohort) and 53% (in EuroILC cohort) of ILC metastases harboured at least one mutation previously associated with endocrine resistance [38]. Further, alterations private to the metastasis compared to the paired primary ILC (of 32 patients) were seen in such genes, including mutations in *CDH1*, *ARID1A*, *ERBB2*, *ESR1*, *AKT1*, *GATA3*, and *NF1*; copy number deletions for *MAP2K4*, *NCOR1*, *TP53*, *PTEN*, and *AKT1*; and amplifications of *CCND1* and *CCNE1*. ILCs from EuroILC and MSK-IMPACT were in general similar, whilst there was an increase in *CDH1*, *ERBB2*, *FOXA1*, and *TBX3* mutations and fewer *TP53* mutations between ILC and IBC-NST metastases. Interestingly, an increase in *IGFR1* mutations was specifically noted for the EuroILC ILC metastasis cohort compared to the MSK-IMPACT IBC-NST cohort, and previous findings of increased *NF1* alterations in ILC metastases [40, 78] were not replicated.

In other recent studies, *MDM4* was found to be amplified in 17% of ILC, and in vitro studies confirmed that this negative regulator of TP53 was actually acting in a TP53-independent manner in ILC-like cell lines [42]; *CDK4* amplification was identified as a marker of poor prognostic outcome in early-stage ILC [83]; and somatic mutations in *AR* were identified in some patients developing metastatic ILC in gynaecological sites [18].

Whether these alterations are already present in minor subclones of the primary tumour or arise during treatment exposure, the enrichment of this plethora of targetable and potentially mutations in metastatic deposits of ILC is of high clinical importance. The data not only give a mechanistic explanation for the treatment failure but also provide critical opportunities for next line targeted therapies. This work points to the future for precision oncology applications involving the sampling and sequencing of metastatic lesions, and we await these advances. A cautionary note to this approach is observed when multiple metastases from the same patient are sequenced. This frequently reveals intra-patient, inter-metastasis heterogeneity for gene mutations involved in treatment resistance [18, 78, 84, 85], suggesting there will remain challenges. Whilst sampling all metastatic foci is not a practical solution, sequencing circulating DNA from liquid biopsies may solve some of these issues.

### Molecular prognostication

Prognostication in breast cancer is routinely performed using clinico-pathologic information, namely the Nottingham Prognostic Index (NPI [86]), which comprises tumour size, grade, and lymph node status, and an IHC panel to evaluate ER, PR, and HER2 (with or without Ki67, a marker of proliferation) [87]. This remains challenging for ILC prognostication, as ILCs are particularly homogenous for each of these parameters, most commonly being grade 2, T1 or T2 in size, with relatively uniform expression of ER, PR, and HER2 [3]. There is then little with which to discriminate which ILC may have a poorer outcome than others as most are in the good to moderate NPI category [47], and indeed with the exception of adjuvant endocrine therapy, ILCs are managed clinically as though independent of their histology.

A number of molecular signature-based tests are available commercially [88]; however, the utility of some of these tests in ILC has only recently emerged (Table 3). Notably, none of these signatures account for tumour morphology in their algorithms. The Genomic Grade Index (GGI/MapQuantDx™) panel has been shown to be more powerful than grade alone in the ILC population [89], and interestingly was able to recategorise some PILC to lower risk, and some classic ILC to higher risk groups based on their calculated genomic grade. MammaPrint® initially had validated value only in node negative ILC patients [90], wherein a hazard ration of 11 was calculated for distant metastasis-free survival in the high-risk category. Recent reports of the MINDACT trial data demonstrate that MammaPrint® classed 16% of ILC as high risk, and 38% as genomically low risk, thus adding value to the clinical assessment of risk with respect to preventing overtreatment with chemotherapy [91]. OncotypeDx® is applied to inform decision-making around the application of chemotherapy [101]. The clinical utility in ILC of the 21-gene OncotypeDx®, remains unclear; two studies show classification of 42% [102] and 35.5% [103] of patients into the intermediate-risk group, which is considered challenging to manage clinically [104]. However, a multivariate analysis of the SEER dataset showed that the OncotypeDx recurrence score was an independent prognostic indicator in ILC [96], and a recent analysis of the PlanB trial data determined that OncotypeDx® classed 72% of ILC as intermediate risk, and 20% and 8% as low and high risk, respectively, amounting to a threefold lower rate of high-risk results compared to non-lobular cancers [55]. These low rates of high-risk ILC are similar to those observed elsewhere [97, 98]. Furthermore, Wu et al. recently reported that PR negativity and high grade may be good indicators of ILC warranting OncotypeDx, and that PR+/G1,2 ILCs are unlikely to have a high RS [105]. Prosigna® is the commercial diagnostic test based on the PAM50 ‘intrinsic’ subtyping (long established as being prognostic in breast cancers [106]). The Prosigna® test generates a Risk of Recurrence score (ROR), and the latest research demonstrates that ROR provides additional prognostic value in ILC, with an increased 10-year distant recurrence rate significantly associated with luminal B status [99] (see [6] for a detailed summary of the transcriptomics of ILC). Early indications suggested that EPClin, the EndoPredict test [107], was highly prognostic in ILC, in both lymph node negative and positive patients, and this was recently confirmed by a pooled analysis of 470 ILC patients who received tamoxifen and/or anastrozole via involvement in three phase III clinical trials [100]. We recently developed a 194-gene signature capable of significantly stratifying prognosis in ILC patients (LobSig [108];) using an integrative analysis of genome copy number and the transcriptome data from ILC tumours. LobSig outperformed NPI, Prosigna, OncotypeDx, and Genomic Grade Index in a stepwise, multivariate Cox proportional hazards model, particularly in grade 2 ILC cases (*χ*^2^, *P* = 9.0 × 10^–6^). This is the first gene signature created with a primary focus of prognosticating ILC patients. We reported that ILCs associated with a high-risk score were enriched for mutations in *ERBB2*, *ERBB3*, *TP53*, *AKT1*, and *ROS1*, highlighting the potential application of targeted therapies in the high-risk ILC patients.

**Table 3:** ILC and benefit of genomic companion diagnostic tests

Test	Ref.	Cohort		Results	Study conclusion
**GGI/MapQuantDx™**	[89]	166 ILC		Test outperformed grade	Prognostic value in ILC
**MammaPrint**	[90]	217 ILC		Independent value of MammaPrint, specifically in lymph node-negative ILC	
	[91]	487 ILC (255 CILC)		10.2% CILC and 22.8% of ILC variants were high risk	Prognostic value in ILC
**OncotypeDx**	[55]	353 ILC		20% low-, 72% intermediate-, and 8% high-risk score	ILC more likely low/int score but 5-year DMFS equivalent to non-ILC
	[92]	30 ILC		All ILC low or int risk	Questions utility in ILC; more data required
	[93]	97 ILC		1% of ILC (non-pleomorphic) record high-risk RS	Questions utility in ILC; more data required
	[94]	102 ILC		Different RS distribution in ILC v IBC-NST	More data required
	[95]	59 ILC		50% ILC in low risk	More data required
	[96]	9037 ILC	SEER data	38.1% ILC intermediate risk; 2.4% high risk	More data required
	[97]	7316 ILC	SEER data	72% ILC in intermediate-risk group; 8% high risk	Adjuvant Ctx did not confer survival benefit to int or high risk; note LN+ cases included
	[98]	49,819 ILC	Genomic Health clinical lab 2004–2017	63.9% ILC in low risk, 33.6 in intermediate, 2.5% in high risk	Classic ILCs have lower average RS (16.3) compared to IDC (18.4) and ILC variants (18.2), and lower rate of tumours with high scores (2.5% vs. 10.7% vs. 8.4%, respectively)
**Prosigna**	[99]	341 ILC	Danish Breast Cancer Group	ILC had poorer 10-year DR rates than ROR matched IDC	Prognostic value in ILC
**EndoPredict/EPClin**	[100]	470 ILC	TransATAC and ABCSG-6/8	63.4% were low EPClin risk group (a 10-year DR risk of 4.8%) compared to 172 (36.6%) women in the high-risk group (110-year DR risk of 26.6%)	Significant prognostic value; Ctx in low-risk group not indicated

The challenge for the validation of predictive and prognostic diagnostics in ILC remains that the latency between diagnosis and relapse/recurrence can be long, making prospective studies difficult to fund and follow. This is also evidenced by the sometimes-contradictory data on whether ILC or IBC-NST has a poorer outcome over time. We excitedly await advances in this field, and consider that they will likely have huge impacts on patients.

### Signalling pathways: biology and therapeutic implications from the use of model systems

A number of studies have employed murine models of ILC and ILC-like cell lines (for example, MDA-MB-134-VI, SUM44PE, and BCK4 [109]), to make advances in understanding the mechanisms underpinning the peculiar biology of ILC and potential therapeutic opportunities. SUM44PE was shown to have acquired a naturally occurring *ESR1* mutation following long-term oestrogen deprivation [110], and this represents an excellent model with which to further address issues surrounding endocrine therapy resistance, which is an increasing clinical problem. In an effort to understand the tamoxifen resistance often seen in ILC, Stires and colleagues identified that resistant ILC cell models had alterations to the MAPK and metabotropic glutamate receptor signalling pathways; these could be pharmacologically targeted [111] to subvert this acquired cellular protection mechanism.

In cell lines grown under oestrogen-deprived conditions, it was found that WNT4 is driving a novel signalling mechanism that modulates ER response [112], and in a recent update, it was demonstrated that WNT4 mediates mTOR signalling through the phosphorylation of S6 kinase [113] whilst also suppressing MCL1, thus influencing metabolic function. Work in the BCK4 cell line and corresponding xenografts showed that c-Kit regulates oestrogen-dependent proliferation, thus implicating c-Kit as a therapeutic target in ILC, with pre-clinical data demonstrating that imatinib (Gleevec®) inhibits proliferation of ILC-like cell lines [114]. The presence of oestrogen has also been shown to activate distinct signalling pathways in ILC (compared to IDC), including PI3K/Akt/mTOR signalling, in part through the actions of WNT4 [112]. ILC-like cell lines were reported to have increased proliferation (compared to IDC lines) and induction of PI3K/Akt/mTOR signalling when cultured under Ultra Low Attachment (ULA) conditions, mimicking anoikis-independence. Interestingly, these cells were not sensitive to PI3K/mTOR dual inhibitors [115].

Similarly, and further to the idea that FGFR1 might be a driver of ILC [116], FGFR4 was identified as a candidate molecule involved in ER resistance in vitro, with clinical relevance to this finding being revealed through *FGFR4* upregulation and mutations being subsequently found in metastatic ILC [117]. FGFR2 was also identified as a key driver of ILC using transposon mutagenesis in mouse mammary models [118], and despite initial sensitivity, tumours became resistant to FGFR inhibitor AZD4547. These resistant tumours showed the acquisition of secondary mutations in *FGFR2*, overexpression of MET, increased drug efflux activity, and inactivation of negative regulators of RAS signalling [119]. Whilst a number of pieces of evidence support insulin-like growth factor-1 receptor (IGF1R) as a promoter of mammary tumorigenesis, clinical trials of IGF1R inhibitors showed responses only in a proportion of cases. It is now clear from the work of Nagle et al [120] that a loss of E-cadherin (a canonical feature of ILC) hyperactivates the IGF1R pathway, whilst also sensitising to anti-IGF1R small-molecular inhibitor therapies OSI-906 and BMS-754807. Pre-clinical evidence to support a role for *IRS2* mutations in driving PILC invasion has recently been presented [37], further implicating the insulin receptor (IR)/IGF1R/IRS2 signalling pathway in PILC biology.

In a murine model of classic ILC (*Cdh*^*Flox/Flox*^*;Pten*^*Flox/Flox*^; loss of E-cadherin and PTEN), inhibition of PI3K signalling with BEZ235 resulted in tumour regression [121]. An alternate mouse model of ILC (*Keratin14*-cre;*Cdh1*^*Flox/Flox*^*;Trp53*^*Flox/Flox*^) was used to demonstrate temporary therapeutic benefit of the mTOR inhibition (AZD8055), leading to pathway suppression and tumour response that was mediated, at least in part, by the adaptive immune response [121]. Murine genetic screens using transposon mutagenesis facilitated the identification of truncating mutations in *ASPP2* (*Trp53bp2*) as a novel oncogenic driver in ILC [118]. It has since been shown that this variant is involved in the initiation and progression of ILC growth, dependent on its cooperative interactions with the actin cytoskeleton, E-cadherin loss, and PI3K activation through PTEN loss [122].

Pre-clinical studies have found that SREBP1 and FASN, and lipid metabolism more broadly, may play key roles in the acquisition of resistance to aromatase inhibitors in ILC [123], raising the possibility of targeted therapies to prevent resistance. Genetic screens to investigate synthetic lethality in the context of E-cadherin deficiency identified the loss of tyrosine kinase ROS1; pre-clinical work supports the administration of foretinib and crizotinib to elicit a tumour killing effect [124], and phase II trials are underway (see the ‘Clinical trials in ILC’ section below). In an effort to identify clinically actionable pathways downstream of E-cadherin loss, Derksen and colleagues used CRISPR/Cas9 knockouts to show increased growth factor receptor (GFR)-dependent activation of PI3K/Akt signalling; treatment with Akt inhibitors resulted in robust inhibition of tumour growth in their murine ILC models [125].

Although ER-positive IBC-NST and ILC are treated similarly, the long-term survival of patients implies that the endocrine therapy response differs between the cancer types. ILC tumours show discordance between *ESR1* mRNA levels and ERα and in fact have alternate ER signalling pathways [126]. Sreekumar et al. [127] showed that two Selective Oestrogen Receptor Downregulators (SERDs) had different effects in ILC-like cell lines, including less effective ERα destabilisation and inefficient suppression of proliferation.

With these now well-established pre-clinical ILC models, we can expect to see continued advances investigating the sensitivities of ILC to a range of targeted therapies.

### Clinical trials in ILC

ILC is now well-established as a distinctive disease process, and increasing clinical evidence supports that a ‘one size fits all’ approach to therapy for all invasive breast carcinomas is not optimised for special subtypes such as ILC. Thus, a number of clinical trials designed to investigate improvements to the therapeutic management of ILC have emerged. Whilst most ILCs are hormone receptor positive and amenable to treatment with endocrine therapies, there has been little investigation into the optimisation of adjuvant endocrine therapy. ‘Endocrine response in women with invasive lobular carcinoma’ (NCT02206984) is an example of a clinical trial looking to optimise a targeted approach to endocrine response in ER+ positive ILC in postmenopausal women [128]. Patients are to be treated with different neoadjuvant anti-oestrogenic therapies, either fulvestrant, anastrozole, or tamoxifen. Changes in Ki67 staining are being utilised as a surrogate marker for outcome and will be correlated to changes in the expression of ER and ER-related genes [128]. The aims of this study include determining whether aromatase inhibitor resistance or increased fulvestrant sensitivity is present within ILC with the intention of further refining optimal adjuvant endocrine-based treatment in this patient cohort.

Cyclin-dependent kinase inhibitors (CDK4/6 inhibitors; e.g. palbociclib) are approved for use in the setting of ER+ metastatic breast carcinoma and prolong survival in combination with fulvestrant [129] and there is a current open-label phase II trial (PELOPS; NCT02764541) investigating the effect of neoadjuvant palbociclib in combination with endocrine therapy in hormone receptor positive early stage breast cancer [130]. This trial also has an initial ‘window phase’ aimed at examining whether tamoxifen or letrozole is more effective in treating ILC, prior to the ‘treatment phase’ wherein patients are randomised to endocrine therapy with or without palbociclib, with the endpoint examining for pathological complete response in the tumour [130]. Clinical trials recognising the unique molecular profile of ILC and the emergence of new therapeutic targets are very encouraging steps toward more tailored treatment strategies for ILC patients, and we await final outcome data on these studies.

Recent studies have indicated a subset of ILC with increased lymphocytic infiltration may be responsive to immunotherapies [68, 70]. Currently, a small phase II trial (GELATO; NCT03147040) is investigating the response of metastatic ILC to combined chemotherapy (carboplatin) and immunotherapy with a monoclonal antibody to Program Death Ligand 1 (PD-L1 inhibitor; atezolizumab) [131]. The prevalence of *ERBB2* mutations in ILC and their responsiveness to neratinib has been confirmed in the MutHER (NCT01670877) trial [45], with this study expanding to include fulvestrant in future phases.

ROLO (NCT03620643) [131] is a phase II trial targeting patients with advanced ILC and aimed at exploiting synthetic lethality, namely the interaction of E-cadherin loss and inhibition of the tyrosine kinase ROS1 [124]. Eligible patients with advanced E-cadherin defective ILC are treated with crizotinib, a readily available ROS1 inhibitor, in combination with fulvestrant [132]. Similarly, the ROSALINE (NCT04551495) phase II trial will investigate entrectinib, a potent ROS1 inhibitor, in combination with letrozole [133]. Another novel therapy being trialled in the setting of metastatic ILC is eribulin, an anti-mitotic single chemotherapeutic agent which affects microtubules within tumour cells [134]. Retrospective analysis of three clinical trials has shown eribulin has similar efficacy in advanced ILC patients who have previously received taxanes and anthracyclines to IDC, suggesting this may represent an alternative systemic chemotherapeutic agent in the metastatic ILC setting [134].

The clinical trial space for ILC has increased rapidly in the last 5 years, with targeted and chemotherapies being evaluated. Additionally, new combinations and treatment sequencing strategies are also being assessed, often as a consequence of resistance identified in previous trials (e.g. PELOPS to resolve tamoxifen resistance identified in BIG1-98/ABCSG-8 [135]).

## Concluding remarks

As research to better understand the lobular subtype of breast cancer intensifies, it is increasingly clear that the distinct morphology, biology, and clinical manifestations of ILC necessitate specialised solutions for management. It is our hope that the next 5 years will deliver on this, off the back of the impressive foundations laid.

## Data Availability

Not applicable
